# The validity of reticulocyte hemoglobin content and percentage of hypochromic red blood cells for screening iron-deficiency anemia among patients with end-stage renal disease: a retrospective analysis

**DOI:** 10.1186/s12882-020-01796-8

**Published:** 2020-04-22

**Authors:** Nhan Hieu Dinh, Suzanne Monivong Cheanh Beaupha, Loan Thi Anh Tran

**Affiliations:** 1 Department of Pharmacology, University of Medicine and Pharmacy at Ho Chi Minh City, Ho Chi Minh City, Vietnam; 2 Department of Internal Medicine, University of Medicine and Pharmacy at Ho Chi Minh City, Ho Chi Minh City, Vietnam; 3 Department of Hematology, University of Medicine and Pharmacy at Ho Chi Minh City, Ho Chi Minh City, Vietnam; 4 Department of Hematology Laboratory, Cho Ray Hospital, Ho Chi Minh City, Vietnam

**Keywords:** Chronic kidney disease, Renal failure, Anemia, Iron, CHr, Hypo

## Abstract

**Background:**

Early detection of iron-deficiency anemia (IDA) can enhance the efficiency and effectiveness of clinical treatment and quality of life for end-stage renal disease (ESRD) patients. This study aimed to evaluate the validity of CHr and %Hypo in screening IDA among ESRD patients and compare their performance with screening IDA among non-ESRD patients.

**Method:**

A retrospective analysis of 312 participants was conducted at Cho Ray Hospital, Vietnam, including healthy control participants and ESRD patients. Receiver operator characteristics curves and the area under the curve (AUC) of models were used to evaluate the performance of CHr, %Hypo. Optimal cut-off values were determined using Youden’s index.

**Results:**

Detecting IDA in ESRD patients is more complicated, as the screening performance of CHr and %Hypo in predicting IDA among ESRD patients were lower than non ESRD group, but still reasonable with AUC = 0.748 (95% CI: 0.656–0.840, power = 0.997) and 0.740 (95% CI: 0.647–0.833, power = 0.996), respectively. Cut-off values of CHr < 31.5 pg and %Hypo> 10.0 pg are recommended to obtain optimal screening ability for Vietnamese ESRD patients.

**Conclusion:**

CHr and %Hypo appears to be useful tools for screening IDA among both non ESRD and ESRD patients. The low cost and accessible of the two markers encourage their utility as effective screening tools in clinical practice.

## Background

Anemia is a common complication among patients with chronic kidney disease (CKD), which occurs in over 50% of non-dialysis-dependent patients [1]. The severity of anemia is intimately associated with the stage of kidney damage, of which up to 75.5% of patients with end-stage renal disease (ESRD) are presented with anemia [2]. Insufficient production of erythropoietin due to kidney failure and iron deficiency are the most common factors causing anemia among CKD patients [1]. On average, an ESRD patient on hemodialysis treatment is estimated to lose ≥2000 mg of iron per year due to several reasons such as occult blood loss, nutritional conditions, infection, systemic inflammatory conditions or remaining blood in the dialyser equipment [1]. Early detection of iron-deficiency anemia (IDA) can significantly enhance the effectiveness of clinical treatment which results in better patient outcomes, reduce treatment cost and improve their quality of life [3].

The most commonly used test to diagnose anemia is hemoglobin, a subtance derived from all types of red blood cells from early progenitors to mature erythrocyte with an average lifespan of around 120 days, which makes the impacts of iron deficiency on hemoglobin requires significant time to be observed [4]. On the other hand, storage of iron in bone marrow is the current gold standard to examine iron status [5]. Obtaining this index requires bone marrow biopsy, an invasive procedure which is impossible to apply in routine practice, thus indicating iron often rely on indirect indices such as serum concentrations of iron, ferritin, or transferrin saturation (TSAT) [6]. Although they are the most familiar to physicians hence had been widely used, concerns had been raised that these markers do not ensure correlation with bone marrow iron stores or hemoglobin response to iron [7]. Moreover, in resource-limited developing countries, ferritin and TSAT are not available in all facilities, especially small hospitals and community health center.

Several alternative markers have been proposed in recent years, of which reticulocyte hemoglobin content (CHr) and percentage of hypochromic red blood cells (%Hypo) are the most promising [8–11]. The reticulocytes last only 24 to 48 h in circulation before developing into mature red blood cells. During the initial stages of iron deficiency, insufficient iron supply would causes a decline of hemoglobin production in reticulocytes in bone marrow, which can be detected through reticulocyte hemoglobin content [4, 12]. Indeed, measuring CHr provides more updated information about functional state of the bone marrow iron status, the development, progression and response to treatment of iron deficiency [13]. Percentage hypochromic red blood cells is a parameter to indicate the percentage of red blood cells contains low level of haemoglobin. Not only an indicator of iron restricted erythropoiesis, %Hypo can also reflect the functional iron status several months before early clinical manifestations of anaemia can be observed. A research from Tessitore et al. had shown that %Hypo is even more accurate than ferritin, TSAT and CHr in predicting IDA among hemodialysis patients [14].

Similar to other developing countries, ferritin and TSAT have not been available in all hospitals and might cause additional economic burden for patients in Vietnam. Meanwhile, CHr and %Hypo cost only one-fourth as much as other haematological parameters and are currently available on many hematology analyzers which does not cause additional payments [12]. Utilization of the two parameters would be beneficial to population in developing countries with resource-limited settings. While studies have shown CHr and %Hypo as efficient predictor for iron deficiency, no consensus was found for a global threshold [15]. Cut-off values of CHr and %Hypo vary according to study population, previous studies had suggest to determine specific cut-off for each target population [15], but the available cut-off values are not validated in Vietnamese population. This study aimed to evaluate the performance of CHr and %Hypo in screening iron deficiency anemia among ESRD patients in comparison with non ESRD group. We also investigated the distribution of CHr and %Hypo in different groups of patients to further examine whether a fixed cut-off value can be applied to predict IDA in normal population and ESRD patients.

## Methods

### Study design

This is a retrospective analysis of 312 participants conducted at Cho Ray Hospital, Ho Chi Minh City, Vietnam from December 2016 to March 2017. According to the hospital routine, participants were collected 2 ml blood anticoagulated with EDTA to performed conventional hematology tests including hemoglobin, RBC, WBC, PLT, MCV, MCH, MCHC, CHr, %Hypo, anemia patients were subsequently examined with ferritin, transferrin and transferrin saturation (TSAT) to measure iron status. All hematology tests were measured using an automated hematology analyzer ADVIA 2120i (Siemen Medical Solutions Diagnostic, USA). Participants were selected using their medical records from the hospital database based on particular inclusion and exclusion criteria and divided into four specific groups for analysis.

Healthy or non ESRD control group included participants who were at least 18 years old, taken medical examination for job or immigrant application, without any chronic disease confirmed by doctors from Department of Internal Medicine, with estimated glomerular filtration rate (eGFR) ≥60 ml/min/1.73m^2^. In terms of laboratory parameters, healthy participants without diagnosis of anemia were defined by Hb ≥12 g/dl in women or ≥ 13 g/dl in men, MCV ranging from 80 fl–100 fl, MCH ≥24 pg, MCHC ≥315 g/l, WBC < 10 g/l and PLT > 150 g/l. Non ESRD patients with IDA were selected from the control group, with anemia was defined by Hb < 12 g/dl in women or < 13 g/dl in men, and iron deficiency diagnosed based on ferritin < 15 ng/ml [16].

ESRD cases were collected from Department of Nephrology, Dialysis and Transplantation. Inclusion criteria were participants aged 18 years and above with kidney failure based on eGFR < 15 ml/min/1.73 m^2^ according to KDIGO 2012 [17]. Patients who had received iron therapy or blood transfusion within the last 3 months or had been diagnosed with thalassemia were excluded from the study. Anemia was defined by Hb < 12 g/dl in women or < 13 g/dl in men. Iron deficiency anaemia was defined as Hb < 12 g/dl in women, < 13 g/dl in men, TSAT < 20%, ferritin < 100 ng/ml.

### Statistical analysis

Continous data are presented as mean value ± standard deviation (SD), or median with interquartile range (IQR). Categorical data are described as frequency and percentage. Quantitative variables were analysed with Student’s t test or Wilcoxon rank sum test as appropriate. For comparison of categorical data, the chi-squared test or Fisher’s exact test was performed. Pearson correlation coefficients were computed to indentify the correlation of CHr and %Hypo with other haematological indices. Results were considered significant with two-sided *p*-value < 0.05.

Logistic regression model was built to determine the association between CHr, %Hypo and IDA. Receiver operator characteristics (ROC) curves and the area under the curve (AUC) of models were used to evaluate the performance of CHr, %Hypo and their combination in screening IDA over control and ESRD group using ferritin and TSAT as the reference. Power of AUC at significant level 0.05 were reported. Optimal cut-off values for the two markers were determined using Youden’s index. Cut-off values were validated with a public dataset of South African patients available from Nalado et al. [15].

All statistical analyses were performed using R version 3.6.1.

### Ethical issues

This study has been approved by the Biomedical Research Ethics Committee at the University of Medicine and Pharmacy at Ho Chi Minh City, Vietnam (Approval number: 415/DHYD-HD, December 1, 2016).

## Results

Table 1 shows the characteristics and laboratory data over four groups of participants. Most ESRD patients were over 50 years old, while the majority of healthy and IDA patients were under 40 years old. The percentage of female in IDA, ESRD non-IDA and ESRD IDA group were higher than male, while they were relatively equivalent in the healthy group.

**Table 1:** Characteristics of participants

Characteristics	Healthy (***n*** = 145)	IDA (***n*** = 59)	Non-IDA ESRD (***n*** = 50)	IDA ESRD (***n*** = 58)
**Age (years)**				
Mean ± SD	36 ± 10.8	39.7 ± 10.8	50.3 ± 15.9	53.7 ± 16.8
< 30	46 (31.7)	13 (22.0)	5 (10.0)	3 (5.2)
30–40	61 (42.1)	16 (27.1)	11 (22.0)	12 (20.7)
41–50	22 (15.2)	23 (39.0)	6 (12.0)	9 (15.5)
51–60	11 (7.6)	5 (8.5)	11 (22.0)	20 (34.5)
> 60	5 (3.4)	2 (3.4)	17 (34.0)	14 (24.1)
**Gender, n (%)**				
Male	72 (49.7)	14 (23.7)	17 (34.0)	21 (36.2)
Female	73 (50.3)	45 (76.3)	33 (66.0)	37 (63.8)
**Hb (g/l)**				
Mean ± SD	144.5 ± 12.3	90.4 ± 18.4	96.2 ± 18.1	92.7 ± 17.5
**CHr**				
Mean ± SD	31.2 ± 1.2	23.4 ± 3.2	31.5 ± 1.9	29.3 ± 2.8
**%Hypo**				
Median (IQR)	2.1 (1.3–3.6)	30.9 (14.0–54.0)	4.2 (1.4–7.6)	9.2 (4.4–18.3)
**MCHC**				
Mean ± SD	330.2 ± 8.2	302.7 ± 23.7	322.3 ± 10.8	315.8 ± 13.6
**MCV**				
Mean ± SD	91.3 ± 4.1	70.6 ± 9.9	92.3 ± 4.8	88 ± 8.8
**Ferritin (ng/ml)**				
Median (IQR)		6.0 (4.2–9.5)	547.5 (358.1–891.5)	339.5 (108.3–744.6)
**TSAT (%)**				
Median (IQR)			30.0 (25.7–36.5)	11.0 (6.7–16.1)
**Transferrin (ng/ml)**				
Median (IQR)			174.9 (151.3–199.3)	170.0 (144.1–195.3)

**Fig. 1:**
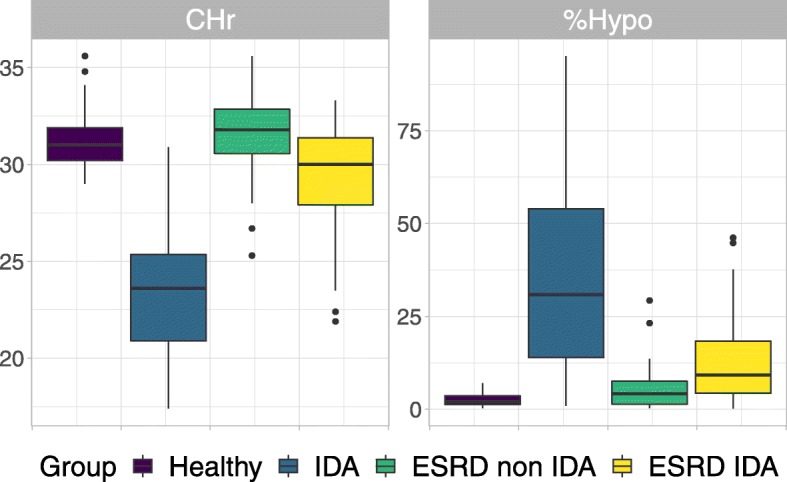
Distribution of CHR and %Hypo among groups of participants

Among groups of participants, IDA patients had the lowest CHr and highest %Hypo (Fig. 1). CHr of IDA cases were lower than non-IDA among ESRD patients (*p* < 0.001) and healthy control group (p < 0.001). %Hypo of IDA were also significantly higher than IDA cases among ESRD patients (< 0.001) and control group (*p* < 0.001). Interestingly, CHr and %Hypo between non ESRD IDA patients and ESRD IDA patients are significantly different (both have p < 0.001).

Significant correlation coefficients were found between CHr, %Hypo and hematological parameters among both ESRD patients and control participants, as shown in Table 2. %Hypo showed negative correlations with Hb, MCV and MCHC, while CHr showed positive correlations.

**Table 2:** Correlation of CHr and %Hypo with other hematological parameters among ESRD patients and non ESRD participants

Test	ESRD patients				Non ESRD patients			
	CHr		%Hypo		CHr		%Hypo	
	α	p-value	α	p-value	α	p-value	α	p-value
**Hb**	0.287	0.002	−0.265	0.004	0.859	< 0.001	−0.821	< 0.001
**MCV**	0.806	< 0.001	−0.297	0.001	0.925	< 0.001	− 0.794	< 0.001
**MCHC**	0.482	< 0.001	−0.562	< 0.001	0.723	< 0.001	−0.823	< 0.001

**Fig. 2:**
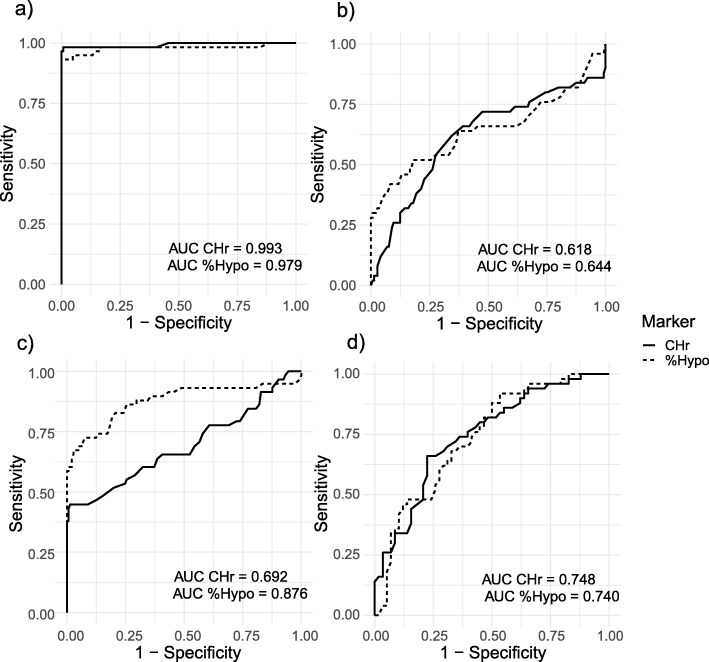
Receiver Operating Characteristic curves for CHr and %Hypo. **a** Discriminating IDA patients from healthy control. **b** Discriminating non IDA ESRD patients from healthy control. **c** Discriminating IDA ESRD patients from healthy control. **d** Discriminating non IDA from IDA among ESRD patients

To detect IDA among non ESRD participants, CHr had AUC = 0.993 (95% CI: 0.978–1.007, power = 1.000), %Hypo has AUC = 0.979 (95% CI: 0.949–1.008, power = 1.000) (Fig. 2a). Combination of CHr and AUC had AUC = 0.991 (95% CI: 0.973–1.000, power = 1.000). The optimal cut-off value for CHR is 29.4 pg for sensitivity of 99.3% and specificity of 98.3%; %Hypo is 7.2 pg for sensitivity of 91.5% and specificity of 100.0%.

In comparison with healthy control group, CHr can discriminate non-IDA ESRD patients with AUC = 0.618 (95% CI: 0.517–0.719, power = 0.725), the AUC of %Hypo was 0.644 (95% CI: 0.537–0.751, power = 0.877). Combination of CHr and %Hypo increased the AUC to 0.732 (95% CI: 0.641–0.822, power = 0.999). The optimal cut-off value for %Hypo is 4.2 pg, which results in 52.0% sensitivity and 82.1% specificity. Likewise, CHr can discriminate IDA ESRD patients from healthy control group with AUC = 0.692 (95% CI: 0.599–0.784, power = 0.993), AUC of %Hypo was 0.876 (95% CI: 0.809–0.943, power = 1.000). A combination of CHr and %Hypo had AUC = 0.876 (95% CI: 0. 809–0.944, power = 1.000). The optimal cut-off value for CHr is 29.5 pg for sensitivity of 98.6% and specificity of 43.6%; %Hypo is 5.2 pg for sensitivity of 72.4% and specificity of 92.4%.

Among anemia ESRD patients, CHr can discriminate IDA and non-IDA cases with AUC = 0.748 (95% CI: 0.656–0.840, power = 0.997), similar to %Hypo with AUC = 0.740 (95% CI: 0.647–0.833, power = 0.996). A combination of two markers increases the AUC to 0.776 (95% CI: 0.690–0.862, power = 1.000). The optimal cut-off value for CHr is 31.5 pg with sensitivity of 66.0% and specificity of 77.6%. The optimal cut-off value for %Hypo is 10.0 pg which returns sensitivity of 46.6% and specificity of 92.0%. Using both markers yields a sensitivity of 79.3% and specificity of 64%, with a positive predicted value (PPV) of 71.9%.

Public dataset from Nalado et al. [15] comprises of 42 IDA ESRD and 11 non-IDA ESRD patients was used to validation the identified cut-off thresholds. Combination of the two markers has sensitivity = 95.3%, specificity = 18.2%, and PPV = 81.6%.

## Discussion

Several studies have proposed CHr and %Hypo as promising markers to detect IDA. However, the performance of these tests in ESRD patients and Vietnamese population have yet to be validated. In this study, we investigated the performance of CHr and %Hypo in screening IDA among ESRD patients and compared them with non ESRD population to provide evidence of their utility in resource-limited settings.

Among non ESRD control participants, high correlation between CHr, %Hypo and Hb, MCV, MCHC suggested that CHr and %Hypo are likely to have the same effect with conventional markers on screening anemia. The correlation coefficients in ESRD patients were significant and followed a similar trend, although their magnitudes were less than those in the control group. The findings were consistent with results from Nalado et al. [15]. Likewise, difference in the distribution of CHr and %Hypo were more significant between IDA patients and healthy participants, which resulted in better performance of the two tests in predicting IDA with AUC up to 0.979 and 0.993, respectively. We found that sensitivity and specificity for CHr were 99.3 and 98.3%, respectively, at a cut-off value of 29.4 pg. This performance is higher than finding from Mustafa et al. with a cut-off of 29 pg which showed sensitivity and specificity at 90.6 and 66.7% [18]. In terms of %Hypo, our suggested cut-off is 7.2 pg for sensitivity of 91.5% and specificity of 100.0%.

Detecting IDA in ESRD patients is more complicated due to the impact of underlying inflammatory condition which resulted from several factors such as uremia, infections, the presence of indwelling dialysis catheters and comorbidities [5]. Indeed, results have shown that unlike in the control group, the AUC of CHr to predict IDA in ESRD patient was only 0.692. However, %Hypo alone can discriminate IDA ESRD patients from healthy control group with AUC = 0.876 (95% CI: 0.809–0.943). This result is consistent to Tessitore et al. [14], which also indicated %Hypo as the most accurate markers to predict response to intravenous iron. The European best practice guidelines has recommend using either %Hypo, CHr, or TSAT to detect functional IDA [19]. One caution when using %Hypo is that the corpuscular hemoglobin concentration can be degraded over time, a phenomenon known as red blood cell swelling, therefore the accuracy of this test requires short storage time of blood sample. However, unlike in the United States where laboratories are national centralized which increased transportation and storage time, laboratories in Vietnam are local and have short storage time just as in Europe, the utility of %Hypo is feasible and reasonable [12].

The AUC of CHr and %Hypo to predict ESRD non-IDA patients were low, indicating the two parameters are specific markers for iron status rather than other causes of anemia. Among anemia ESRD patients, CHr and %Hypo can discriminate IDA and non-IDA cases with AUC = 0.748 and 0.740, respectively. A combination of the two markers can increase the AUC to 0.776 (95% CI: 0.690–0.862), which is consistent to a study from Nalado et al. [15] in which the AUC was 0.76. Unlike Nalado et al. [15] concluded that using CHr alone is sufficient to detect IDA in their study population, our results showed that combining CHr and %Hypo can improve the accuracy, though not much, in comparison with a single test.

Studies had shown that the performance of CHr and %Hypo in detecting IDA among early stages CKD patients (stages I and II) are better than later stages (IV and V), thus specific cut-off values should be proposed to different stages of CKD [15, 20]. Consistent with Kim et al. [21] cut-off of 32 pg to determine IDA among haemodialysis patients, our results found that the CHr cut-off value of 31.5 pg is optimal for Vietnamese ESRD patients. In terms of %Hypo, the cut-off value was 10.0 pg, which is consistent to common recommendations on this parameter [19, 22, 23]. In our validation experiment, although the combination of two markers only has specificity of 18.2%, high sensitivity of 95.3% and PPV of 81.6% was observed which confirmed its performance as an adequate screening tool for IDA among ESRD patients.

Conventional iron tests such as ferritin or TSAT cost around $25–$30, while CHr and %Hypo is significantly cheaper with only $5–$7 and can be performed on several common hematology analyzers [15]. Using CHr and %Hypo as screening tools for IDA would reduce economic burden for ESRD patients, which is especially important for patients in resource-limited countries.

### Study limitations

The main limitation of this study was its retrospective design, which may be subject to bias. Due to lack of resources, iron deficiency anemia was defined using serum ferritin and TSAT as reference instead of the gold standard bone marrow aspiration. As ferritin and TSAT are widely used and accepted in current clinical practice, we believe that our study design and results are valid for the purpose of validating screening tools for IDA. Sample size was small and the study was conducted on Vietnamese patients only, which may limit the capacity of generalising our findings. However, our validation experiment on a public data has shown that the markers have high sensitivity and PPV, which are acceptable for the screening purpose.

## Conclusions

In conclusion, CHr and %Hypo appear to be useful tools for screening IDA among both non ESRD and ESRD patients. A combination of CHr and %Hypo test showed high sensitivity in detecting IDA among ESRD patients who have anemia with the cut-off values for CHr < 31.5 pg and %Hypo> 10.0 pg.

## Data Availability

The datasets used and/or analysed during the current study are available from the corresponding author on reasonable request.

## Abbreviations

%Hypo: Percentage of hypochromic red blood cells
CKD: Chronic kidney disease
CHr: Reticulocyte hemoglobin content
ESRD: End-stage renal disease
IDA: Iron-deficiency anemia
ROC: Receiver operator characteristics
AUC: Area under the curve
TSAT: Transferrin saturation
